# *Tc-knirps* plays different roles in the specification of antennal and mandibular parasegment boundaries and is regulated by a pair-rule gene in the beetle *Tribolium castaneum*

**DOI:** 10.1186/1471-213X-13-25

**Published:** 2013-06-18

**Authors:** Andrew D Peel, Julia Schanda, Daniela Grossmann, Frank Ruge, Georg Oberhofer, Anna F Gilles, Johannes B Schinko, Martin Klingler, Gregor Bucher

**Affiliations:** 1 Institute of Molecular Biology and Biotechnology (IMBB), Foundation for Research and Technology - Hellas (FoRTH), Nikolaou Plastira 100, GR-70013, Heraklion, Crete, Greece; 2 Blumenbach Institute of Zoology, Georg-August-University Göttingen, Justus-von-Liebig-Weg 11, 37077, Göttingen, Germany; 3 Department of Biology, Friedrich-Alexander University Erlangen, Staudtstrasse 5, 91058, Erlangen, Germany

**Keywords:** *Knirps*, Head gap gene, *Tribolium*, Antenna, Mandible, Intercalary segment

## Abstract

**Background:**

The *Drosophila* larval head is evolutionarily derived at the genetic and morphological level. In the beetle *Tribolium castaneum*, development of the larval head more closely resembles the ancestral arthropod condition. Unlike in *Drosophila*, a *knirps* homologue (*Tc-kni*) is required for development of the antennae and mandibles. However, published *Tc-kni* data are restricted to cuticle phenotypes and *Tc-even-skipped* and *Tc-wingless* stainings in knockdown embryos. Hence, it has remained unclear whether the entire antennal and mandibular segments depend on *Tc-kni* function, and whether the intervening intercalary segment is formed completely. We address these questions with a detailed examination of *Tc-kni* function.

**Results:**

By examining the expression of marker genes in RNAi embryos, we show that *Tc-kni* is required only for the formation of the posterior parts of the antennal and mandibular segments (i.e. the parasegmental boundaries). Moreover, we find that the role of *Tc-kni* is distinct in these segments: *Tc-kni* is required for the initiation of the antennal parasegment boundary, but only for the maintenance of the mandibular parasegmental boundary. Surprisingly, *Tc-kni* controls the timing of expression of the Hox gene *Tc-labial* in the intercalary segment, although this segment does form in the absence of *Tc-kni* function. Unexpectedly, we find that the pair-rule gene *Tc-even-skipped* helps set the posterior boundary of *Tc-kni* expression in the mandible. Using the mutant *antennaless*, a likely regulatory Null mutation at the *Tc-kni* locus, we provide evidence that our RNAi studies represent a Null situation.

**Conclusions:**

*Tc-kni* is required for the initiation of the antennal and the maintenance of the mandibular parasegmental boundaries. *Tc-kni* is not required for specification of the anterior regions of these segments, nor the intervening intercalary segment, confirming that *Tc-kni* is not a canonical ‘gap-gene’. Our finding that a gap gene orthologue is regulated by a pair rule gene adds to the view that the segmentation gene hierarchies differ between *Tribolium* and *Drosophila* upstream of the pair rule gene level. In *Tribolium*, as in *Drosophila*, head and trunk segmentation gene networks cooperate to pattern the mandibular segment, albeit involving *Tc-kni* as novel component.

## Background

The insect head is composed of several segments and a non-segmental anterior region. However, the exact segmental composition of the insect head has long been a matter for debate [1-7]. The posterior gnathocephalon is made up of the mandibular, maxillary and labial segments that each bear a pair of appendages specialized for feeding. The anterior procephalon consists of anterior non-segmental parts and an antennal segment, which is separated from the mandibular segment by an appendage-free segment (the intercalary segment), whose development in insects is significantly delayed, as well as reduced in size.

The genetic mechanisms of head segmentation were first examined in the dipteran fruit fly *Drosophila melanogaster*[3]. Its gnathal segments are patterned by the trunk segmentation gene cascade, involving maternal, gap, pair-rule and segment polarity genes [3,8], while the patterning of the procephalic segments follows a different paradigm [3,9-15]. Whilst segment polarity genes (i.e. *en*, *wg*, *hh*) are involved in establishing these segments, pair-rule genes are not [3,10-15]. Four *Drosophila* head gap genes, *orthodenticle* (*otd*), *empty spiracles* (*ems*), *buttonhead* (*btd*) and *sloppy paired* are expressed in broad overlapping domains in the developing anterior head [9,16]. Mutation of these genes leads to classic ‘gap phenotypes’ - the loss of all the adjacent segments covered by their domains of expression [9,17]. However, mis-expression studies have shown that only *otd* affects segment polarity gene expression when expressed in ectopic domains, and only *ems*, with the help of *btd*, appears to confer identity to head segments [18-20]. Indeed, second order regulators have been identified that operate at levels in between the head gap genes and segment polarity genes: i.e. *collier* and *cap ‘n’ collar*[11,12,21,22].

*Drosophila* exhibits an evolutionary derived mode of head development, in which the larval head is greatly reduced and undergoes ‘head involution’ during which head regions are folded into the body cavity [3]. This situation is far from typical for insects and moreover, the reduced and experimentally inaccessible *Drosophila* larval head has limited the comprehensive identification and analysis of insect head development genes for technical reasons [1].

In recent years the red flour beetle *Tribolium castaneum* has emerged as a powerful genetic insect model system [23] and offers an opportunity to study the genetic and cellular mechanisms underlying the development of a more insect-typical head [1]. As in *Drosophila*, the *Tribolium* gnathal segments appear to be patterned using similar mechanisms to those operating in the trunk, including a central role for pair-rule gene homologues [24-30]. In the anterior head, second order regulators and the segment polarity genes might be relatively well conserved between *Drosophila* and *Tribolium*[27,28,31-35]. However, clear differences have been identified at the level of the head gap genes, and the maternally provided anterior protein gradients that establish their expression domains [1,36-40]. For example, while the *Tribolium* homologue of *orthodenticle* (*Tc-otd*) apparently plays a broadly conserved role as a gap gene during head segmentation in *Tribolium*, it appears to be much more involved in axis formation than its *Drosophila* orthologue [36,41,42]. The expression of the *Tribolium* homologues of *empty spiracles* and *buttonhead* (*Tc-ems* and *Tc-btd*) is limited to single segment wide domains instead of large overlapping domains in the blastodermal head anlagen. *Tc-ems* is required to form parts of the antennal segment only and knockdown of *Tc-btd* does not lead to a head cuticle phenotype at all [36]. This raised the question of what genes might control development of these head regions in *Tribolium*. Work by Cerny *et al.*[43] suggests that the answer to this question is, at least in part, the single *Tribolium* homologue of the *Drosophila* genes *knirps* and *knirps-related*.

The *Drosophila* genes *knirps* and *knirps-related* encode steroid hormone receptor-like transcription factors [44-46]. Ancestrally, the insect *knirps* family consisted of two genes, *eagle* and *knirps-related*, while knirps arose via a recent gene duplication of the *knirps-related* gene in the higher Diptera [47]. At the blastoderm stage, *knirps* and *knirps-related* are expressed in almost identical anterior and posterior domains [45,48-50]. *Drosophila knirps* acts as a canonical gap gene during trunk segmentation [51-53]. In contrast, the anterior mandibular expression domain is not required for head segmentation, since segment polarity gene (i.e. *engrailed*) expression in the head is not affected in embryos that lack both paralogues [49] while a loss of the stomatogastric nervous system is observed [49].

Cerny *et al.*[43] have shown that *Tc-knirps* (*Tc-kni*), the single *Tribolium* homologue of the *Drosophila knirps*-family paralogues, is also expressed in anterior and posterior domains during early development [43]. However, the *Tc-kni* posterior domain is shifted anteriorly relative to its position in *Drosophila*, and knockdown of *Tc-kni* does not lead to a canonical gap phenotype in the trunk, but rather minor defects in the posterior abdomen. The anterior expression domain of *Tc-kni* is largely conserved. In contrast to *Drosophila*, the anterior domain does play an essential role in head patterning: Knockdown of *Tc-kni* leads to loss of both antennae and mandibles [43].

Cerny *et al.*[43] found early loss of *Tc-wg* expression in the antennal segment in *Tc-kni* RNAi while the mandibular domain of *Tc-wg* expression disappeared at a later stage. Further, they showed that *Tc-kni* is not needed for correct *Tc-wg* expression in the intercalary segment. Finally, light abnormalities in the maturation and maintenance of the first pair-rule stripe of *Tc-eve* expression were observed, where the distance between the first segmental *Tc-eve* stripe (in the mandibular segment) and the ocular *Tc-wg* expression domain was reduced in *Tc-kni* RNAi blastoderms [43]. It has remained unclear, however, whether antennal and mandibular segments are deleted completely and whether the intercalary segment is affected.

In this study we examined the effect of knocking down *Tc-kni* on a comprehensive set of genes that mark sub regions of the antennal, intercalary and/or mandibular segments. We show that *Tc-kni* is required for correctly specifying only posterior regions of antennal and mandibular head segments (i.e. the parasegmental boundaries). Interestingly, *Tc-kni* is essential for the initial specification of the antennal parasegmental boundary, while it is required only for the maintenance of the mandibular parasegmental boundary. The intercalary segment does not appear to be affected. Unexpectedly, we find that the trunk pair-rule gene *Tc-even-skipped* is required to set the posterior boundary of the mandibular *Tc-kni* expression. Unlike most RNAi studies, we have good evidence that we were investigating a Null situation due to our finding that *antennaless*, a *Tribolium* mutant arising from an EMS screen [54], is a likely regulatory Null-mutation of *Tc-kni*. Taken together, we show that *Tc-kni* is not a head gap gene, since its mutation does not lead to the complete deletion of several adjacent segments. Further, we provide a model, for how head and trunk patterning mechanisms cooperate to pattern the mandibular segment in *Tribolium*, and how these interactions differ between *Drosophila* and *Tribolium*.

## Results and discussion

### Early *Tc-kni* expression prefigures the appearance of the anterior head anlagen

**Figure 1:**
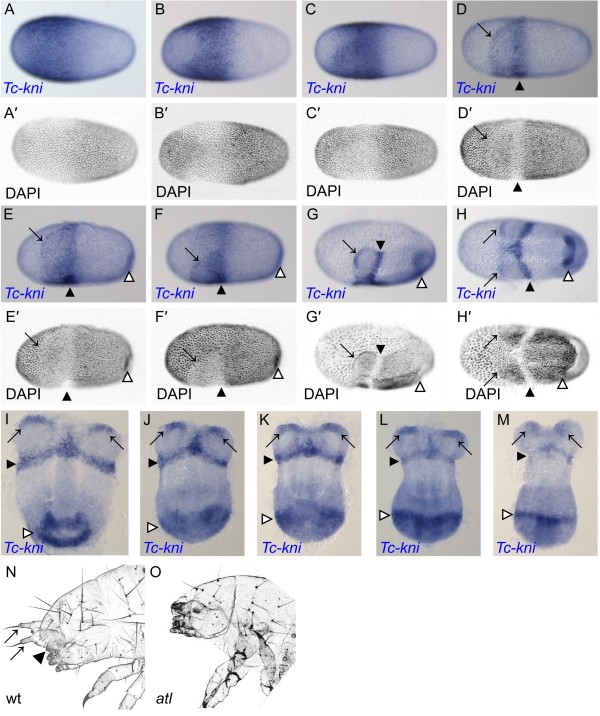
**Embryonic expression of *Tc-kni *and the *Tc-kni *head cuticle phenotype. A-M**: Expression of *Tc-kni* during blastoderm (**A-H**’) and early germband stages (**I-M**). See text for a detailed description. Panels **A’-H’** show inverted DAPI images of the embryos in panels **A-H** respectively. Black arrows in panels **D-M** mark *Tc-kni* expression at the anterior border of one or both head lobes. Black arrowheads in panels **D-M** indicate the stripe of *Tc-kni* expression marking the anterior compartment of the mandibular segment. White arrowheads in panels **E-M** mark the posterior domain of *Tc-kni* expression which first becomes visible within the primitive pit as it forms (**E-I**) and later resolves into a stripe marking segment A1 (**J-M**). Anterior is to the left in panels **A-H**’ and to the top in panels **I-M**. Panel **B** shows a dorso-lateral view, panels **D, E** and **G** show ventro-lateral views, panel **C** and **F** lateral views, and panels H-M are all ventral views. **N-O**: In contrast to wildtype (**N**), *antennaless* mutant first instar larvae (**O**) lack antennae (black arrows in panel **N**) and mandibles (black arrowhead in panel **N**). All lateral and dorsal head bristles are however present.

First, we studied in more detail the dynamics of anterior *Tc-kni* expression because previous studies had focused on posterior aspects [43]. Zygotic *Tc-kni* expression in the syncytial blastoderm begins as a broad domain covering most of the anterior half of the blastoderm, only absent from a small region around the anterior pole (Figure 1A). *Tc-kni* expression then retreats from the anterior pole, but much more so on the dorsal side of the egg, clearing from regions that will later become the extra-embryonic serosa (Figure 1B) [55]. *Tc-kni* expression is maintained in the anterior/ventral regions that will become head tissue [55] (Figure 1C). These early shifts in expression occur before embryonic and extra-embryonic tissue can be distinguished at the level of nuclear morphology (Figure 1A’-C’ and Cerny *et al.*[43]). When this distinction becomes apparent (Figure 1D’-F’), the uniform anterior *Tc-kni* expression domain splits into two anterior lateral domains at the boundary between each head lobe and the abutting extra-embryonic tissue (black arrows), and a more posterior stripe marking the future anterior compartment of the mandibular segment (black arrowheads) (Figure 1D-F). These domains are maintained through subsequent blastoderm (Figure 1G-I) and early germband (Figure 1J, K) stages, before fading (Figure 1L, M) and then disappearing completely during mid-germband elongation stages. Hence, the early uniform domain of *Tc-kni* expression (Figure 1C) encompasses the entire future anterior head anlagen; i.e. everything anterior to the parasegment 0/1 boundary.

### *Antennaless* is likely a regulatory mutation at the *Tc-kni* locus

In most RNAi experiments outside *Drosophila*, it remains unclear to what extent a genetic Null-situation is phenocopied. We aimed at determining the Null situation by studying a genetic mutant.

*Antennaless* is a homozygous lethal mutation that was recovered from an EMS mutagenesis screen [54]. The cuticular phenotypes of *antennaless* mutant larvae are highly reminiscent of *Tc-kni* RNAi phenotypes (*Tc-kni*^RNAi^) [43]. Larvae of the *antennaless* mutant lack antennae and mandibles, and occasionally also display minor abdominal defects, very similar to those previously reported for weak *Tc-kni*^RNAi^ larvae (Figure 1N, O and Cerny *et al.*[43]). In order to check for defects in dorsal and lateral head tissues which are derived from pre-antennal and intercalary regions, respectively [32,36,56], we scored both sides of 20 *antennaless* larval cuticles for the head bristle pattern. All bristles were present, albeit shifted somewhat compared to the wildtype condition in these regions (Figure 1N, O). This is consistent with pre-antennal regions and much of the intercalary segment not being affected in the mutant [32,43].

The development of both mandibles and antennae was more severely affected at a higher temperature in *antennaless* mutants. A larval cuticle was scored as an *antennaless* mutant if either the mandibles or antennae, or both, were reduced or absent. Most of the *antennaless* mutant larvae (67%, n = 43) lacked both the antennae and mandibles at 25°C. This increased to 75% (n = 32) at 32°C. In a few cases, rudiments of mandibles were found (5% at 25°C and 6% at 32°C). In other cases, antennae or antennal rudiments were found (28% at 25°C and 19% at 32°C). In contrast, and against the general observation that phenotypes tend to be more penetrant at higher temperatures, the frequency of abdominal defects was found to be higher at 25°C than at 32°C. At 25°C, 90.5% (n = 43) of phenotypic larvae displayed defects within segments A5-A8, compared to only 16% (n = 32) at 32°C. An inverse sensitivity with respect to temperature was also observed for defects in the urogomphi, dorsal outgrowths of the ninth segment. At 25°C this structure was defective in 79% of the phenotypic larvae examined (n = 43), compared to 44% at 32°C (n = 32).

In offspring from nine independent pairs of heterozygous parents, the *antennaless* cuticle phenotype is found in 25% (n = 364) and 27% (n = 267) of larvae at 25°C and 32°C respectively, levels consistent with a highly penetrant homozygous lethal mutation. A significant proportion of mutant larvae were able to hatch despite their lack of antennae and mandibles; 45% at 25°C (n = 91) compared to only 17% at 32°C (n = 72). The vast majority of these larvae died at the L1 stage. Only very rarely did individuals reach the L2 stage. When corrected for a background reduction in hatching rate associated with this shift in temperature (measured from wildtype larvae), these data reveal a 19% increase in sensitivity to temperature with regards to hatching in the *antennaless* mutant background.

In our *Tc-kni* RNAi experiments head defects were also found to be more severe at 32°C compared to 25°C (see Additional file 1, compare panel B to panel A). The severity of head defects decreased over time post-injection as expected for parental RNAi experiments [57] (see Additional file 1, panel C). However, as for *antennaless*, we found inverse temperature sensitivity with respect to defects in segments A5-A8 following *Tc-kni*^RNAi^ (see Additional file 1, compare panel E to panel D). The frequency of abdominal defects also increased with time post-injection from about 20% (n = 10) at day eight post-injection, to 37% (n = 35) eleven days post-injection, and to 56% (n = 27) thirty-one days post-injection, before dropping again (For eggs at 25°C; see Additional file 1, panel D). This is not in line with the usual observation that in RNAi experiments phenotypic strength decreases over time and indicates a complex relationship between knockdown and phenotype, which we do not fully understand.

Since the *antennaless* cuticle defects described above are almost identical to the *Tc-kni*^RNAi^ phenotypes both in terms of physical phenotype and sensitivity to temperature, we suspected *Tc-kni* as the gene affected by the mutation. We therefore independently sequenced the three *Tc-kni* exons from the genomic DNA of two first instar larvae that had been identified as homozygous mutant by their cuticle phenotype. We found that the coding sequence of *Tc-kni* is not altered in mutant beetles (data not shown).

In order to test the hypothesis that *antennaless* is a regulatory mutation at the *Tc-kni* locus, we carried out *in situ* hybridization on embryos from heterozygous mutant parents with a mix of probes targeting *Tc-kni* and *Tc-caudal* as positive control. We failed to detect *Tc-kni* expression in 15% of the offspring examined (n = 100), whereas *Tc-caudal* was well stained in the same colour reaction in all cases (see Additional file 2). This is consistent with embryos homozygous for the *antennaless* allele not expressing *Tc-kni* at levels detectable via *in situ* hybridization.

Taken together, our results are consistent with the hypothesis that *antennaless* is a regulatory mutation of *Tc-kni*, and as such we now refer to it as *Tc-kni*^atl^. However, we cannot rule out that *antennaless* is a mutation in a gene that acts rather exclusively upstream of, or is a required interaction partner of, *Tc-kni*. Further studies will be necessary to confirm our hypothesis by identifying the cis-regulatory regions that are affected by the mutation.

### The *Tc-kni* Null phenotype is revealed by both *Tc-kni*^atl^ and *Tc-kni*^RNAi^

The lack of *Tc-kni* expression in homozygous *Tc-kni*^atl^ embryos implies that *Tc-kni* function is strongly reduced in the mutant. However, it remains possible that some residual function remains. In order to test whether *Tc-kni*^atl^ constitutes a complete Null phenotype for *Tc-kni*, we performed *Tc-kni* RNAi in the *Tc-kni*^atl^ background, assuming that the added effects should not lead to stronger phenotypes if *Tc-kni*^atl^ is a Null mutant. The frequency of the *Tc-kni* phenotype in the pooled offspring from 30 independent pairs of *Tc-kni*^atl^ heterozygous animals was 11% (n = 149) at 25°C and 17% (n = 82) at 32°C. As expected, after injecting the same females with *Tc-kni* dsRNA, the frequency of the *Tc-kni* phenotype strongly increased, to 97% (n = 32; 25°C) and 81% (n = 32; 32°C). Crucially, we did not find evidence for an increase in the severity of the cuticle phenotype: i.e. there were no larvae with phenotypes more severe than those seen in *Tc-kni*^atl^ or *Tc-kni*^RNAi^ alone. Hence, unlike in most RNAi experiments, we can be rather confident that the most severe RNAi phenotypes we observe fully phenocopy a Null situation. Therefore, we use results from both mutant and RNAi embryos for this work.

### *Tc-kni* is differentially required for specifying antennal, intercalary and mandibular parasegment boundaries

**Figure 2:**
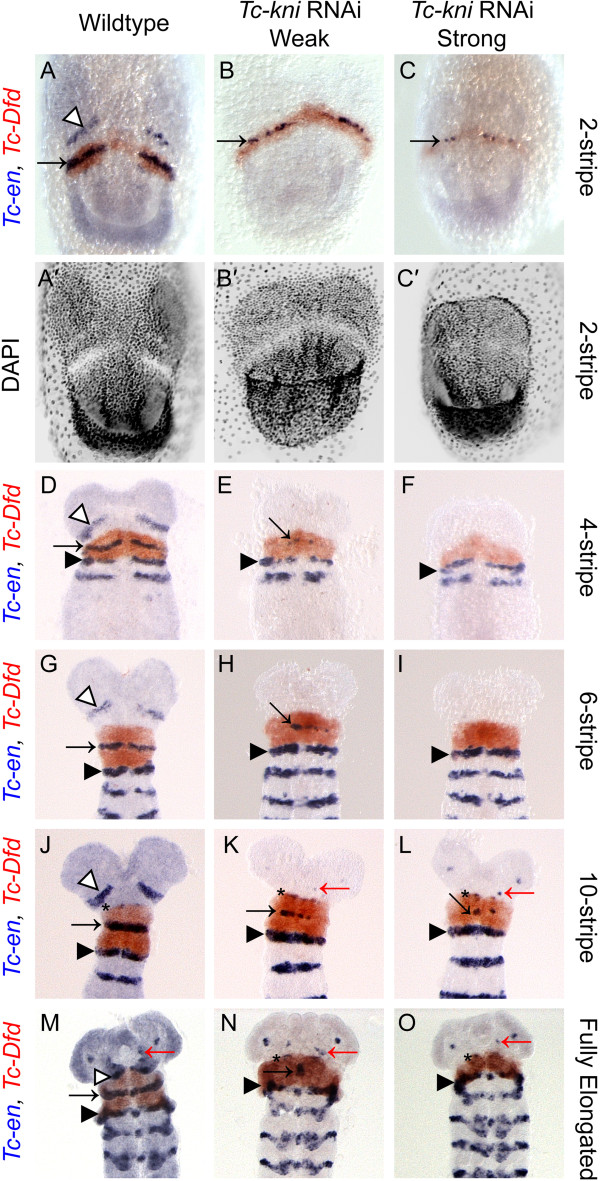
**Expression of *Tc-en *and *Tc-Dfd *in wildtype and *Tc-kni *RNAi embryos.** The antennal *Tc-en* stripe (marked by white arrowhead in wildtype panels **A, D, G, J, M**) is missing in all *Tc-kni* RNAi embryos. The mandibular *Tc-en* stripe (marked by black arrow) forms within the *Tc-Dfd* expression domain, but is broken (panels **B, C, E, H, K, L, N**) and often missing (panels **F, I, O**) in *Tc-kni* RNAi embryos. *Tc-en* expression in the intercalary segment (black asterisk in panels **J-L, N-O**; hidden by antenna in panel **M**) appears at a variable time point in mid-elongation stage embryos at the anterior border of the *Tc-Dfd* expression domain in both wildtype and *Tc-kni* RNAi embryos. *Tc-en* expression associated with structures at the base of the antenna is evident in late *Tc-kni* RNAi embryos (red arrows in panels **K-O**). Head lobes appear reduced in size (panels **A’-C’**), and the *Tc-Dfd* domain slightly narrower (panels **G-O**) in *Tc-kni* RNAi embryos, consistent with a failure to specify and form posterior antennal and mandibular tissue. Black arrowheads in panels **D-O** mark the maxillary *Tc-en* stripe. Ventral views, and anterior towards the top, in all panels.

The cuticle phenotypes reported by Cerny *et al.*[43] raised the question of whether entire segments are deleted in *Tc-kni* depleted embryos, or only the posterior parts that give rise to the appendages. These depend on parasegmental boundary formation via the segment polarity genes *Tc-engrailed* (*Tc-en*), *Tc-hedgehog* (*Tc-hh*) and *Tc-wingless* (*Tc-wg*) [5,10,13,21,22,27-29]. Therefore, we stained *Tc-en* and *Tc-hh* in *Tc-kni*^RNAi^ embryos as markers for the posterior segment compartment. In the case of *Tc-en*, embryos were co-stained for *Tc-Dfd* to help distinguish disrupted mandibular *Tc-en* expression domains (which are located within the *Tc-Dfd* domain), from intercalary *Tc-en* expression domains (arising at the anterior median boundary of the *Tc-Dfd* domain) (Figure 2J-K, N-O, black asterisks).

**Figure 3:**
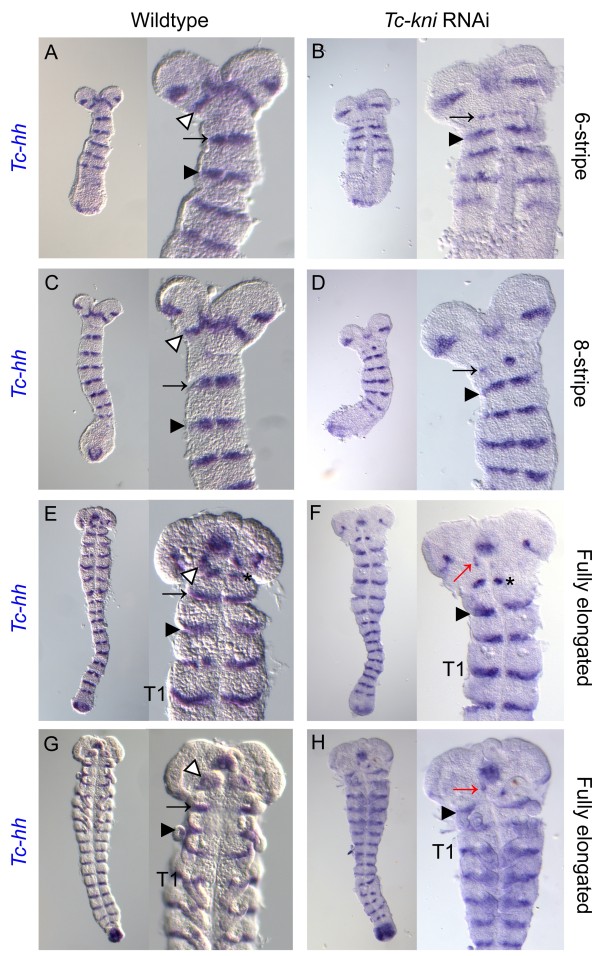
**Expression of *Tc-hh *in wildtype and *Tc-kni *RNAi embryos.** The antennal *Tc-hh* stripe (marked by white arrowhead in wildtype panels **A, C, E, G**) is missing in all *Tc-kni* RNA embryos (panels **B, D, F, H**). The mandibular *Tc-hh* stripe (marked by black arrow in panels **A, C, E, G**) is broken (panels **B, D**) and later missing (panels **F, H**) in *Tc-kni* RNAi embryos. *Tc-hh* expression in the intercalary segment (black asterisks in panels **E, F**) appears at a variable time point in mid-elongation stage embryos in both wildtype and *Tc-kni* RNAi embryos. *Tc-hh* expression associated with structures at the base of the antenna is evident in late *Tc-kni* RNAi embryos (red arrow in panels **F, H**). A black arrowhead in all panels marks the maxillary *Tc-hh* stripe. Ventral views, and anterior towards the top, in all panels.

We find that both *Tc-en* (Figure 2) and *Tc-hh* (Figure 3) expression is absent in the antennal segment in *Tc-kni*^RNAi^ embryos throughout embryogenesis (marked by white arrowhead in wildtype embryos in Figures 2 and 3). Occasionally, *Tc-en* and *Tc-hh* expression associated with structures at the base of the antenna remains in later germband embryos, despite the clear failure of the antenna to form (marked by a red arrow in panels K, L, M, N and O in Figure 2 and panels F and H in Figure 3).

The mandibular stripe of *Tc-en* expression is initiated in early *Tc-kni*^RNAi^ germband embryos, albeit abnormally; the stripe of expression is broken and in extreme cases only patches of expression are seen (compare panels B and C with panel A in Figure 2; mandibular stripe marked by black arrow). In older *Tc-kni*^RNAi^ germband embryos, *Tc-en* expression associated with the mandibular segment is often missing completely (compare panels F, I and O, with panels D, G and M respectively in Figure 2), suggesting a failure to properly maintain *Tc-en* expression in this segment in strong knockdowns of *Tc-kni* expression. Similarly, *Tc-hh* expression in the mandibular segment is greatly reduced in young *Tc-kni*^RNAi^ embryos, but a thin broken stripe is still detected medially in early germbands (compare black arrows in panels B and D to panels A and C respectively in Figure 3). Later, this *Tc-hh* expression disappears in contrast to wildtype (compare panels F and H to panels E and G respectively in Figure 3). Note that *Tc-en* mandibular expression appears to be more sensitive to the loss of *Tc-kni* expression in lateral regions of the germband (Figure 2E, H, K and N).

*Tc-en* and *Tc-wg* expression in the intercalary segment appears relatively late and at a quite variable time point during mid germband elongation in wildtype embryos. Whilst we cannot completely rule out minor disruptions of intercalary *Tc-en/Tc-hh* expression initiation and/or maintenance in *Tc-kni*^RNAi^ embryos, we do detect wildtype expression of these genes in late elongation (i.e. post 10 *Tc-en* stripe embryos) and early fully elongated *Tc-kni*^RNAi^ embryos (black asterisk in panels K, L, N and O in Figure 2 and black asterisk in panel F in Figure 3).

In conclusion, the disruption of *Tc-kni* function leads to the complete failure to initiate segment polarity gene expression in the antennal segment, failure to properly maintain segment polarity gene expression in the mandibular segment, and most likely has no effect on segment polarity gene expression in the intercalary segment [43].

### Molecular markers show that *Tc-kni* is not a canonical head gap gene

**Figure 4:**
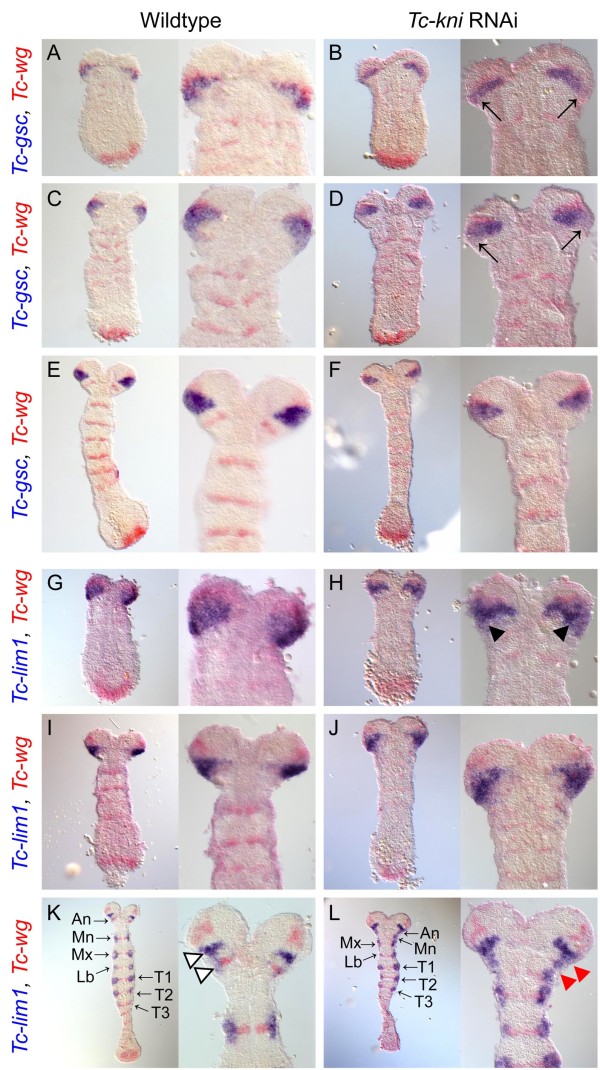
**Expression of *Tc-gsc *and *Tc-lim1 *in wildtype and *Tc-kni *RNAi embryos.** All embryos are also co-stained for *Tc-wg*. *Tc-gsc* expression is reduced in posterior-lateral regions of its normally wedge-shaped ocular/antennal expression domain in early germband *Tc-kni* RNAi embryos (black arrows in panels **B, D**; compare to panels **A, C** respectively). At slightly later germband stages however, clear differences between the *Tc-gsc* expression domain in *Tc-kni* RNAi and wildtype embryos are no longer observed (compare panel **F** to panel **E**). *Tc-lim1* expression is also irregular and reduced in posterior regions of its wedge-shaped ocular/antennal expression domain in early germband *Tc-kni* RNAi embryos (black arrowheads in panel **H**; compare to panel **G**). At slightly older germband stages clear differences in the *Tc-lim1* expression domain are no longer observed between *Tc-kni* RNAi and wildtype embryos (compre panel **J** to panel **I**). In later wildtype germband embryos, the *Tc-lim1* ocular/antennal domain splits into two stripes (marked by white arrowheads in panel **K**). This fails to occur in equivalent stage *Tc-kni* RNAi embryos, and antennal, mandibular and maxillary expression domains are often fused in lateral regions of the germband (red arrowheads in panel **L**; compare to panel **K**). Ventral views, and anterior to the top, in all panels.

Segment polarity genes mark a posterior portion of each segment. Therefore, we also asked whether the anterior parts of the segments are miss-specified and/or missing in *Tc-kni*^RNAi^ embryos by analyzing markers for the anterior regions of head segments. *Tc-goosecoid* (*Tc-gsc*) expression [56] initially partially overlaps the ocular *Tc-wg* domain and extends posterior to it (Figure 4A). Later, this domain widens in lateral regions, forming a wedge shape domain that abuts the antennal *Tc-wg* stripe as it appears (Figure 4C) but later retracts from it (Figure 4E). Thus, *Tc-gsc* is a marker for posterior ocular and anterior antennal regions in early embryos but later predominantly marks ocular tissue. In early *Tc-kni*^RNAi^ germband embryos, *Tc-gsc* expression is down-regulated in posterior-lateral regions of the wedge shaped expression domain (black arrows in Figure 4B, D; compare to Figure 4A, C respectively). In older *Tc-kni*^RNAi^ embryos, the *Tc-gsc* expression domain again closely resembles the wedge shape seen in wild-type embryos at these stages (Figure 4F). This indicates that the ocular parasegment boundary and anterior parts of the antennal segment are not greatly affected by loss of *Tc-kni*, apart from some degree of lateral down-regulation of *Tc-gsc* in early germ bands.

*Tc-lim1* is a similar marker for the posterior of the ocular segment and anterior compartment of the antennal segment [56], albeit shifted slightly posteriorly relative to *Tc-gsc*. In early embryos, expression is partially overlapping the ocular *Tc-wg* domain and extending posterior to it (Figure 4G). In elongating germbands, *Tc-lim1* expression forms a wedge shaped domain that covers all cells between the ocular and antennal *Tc-wg* stripes (Figure 4I). In the *Tc-kni*^RNAi^ background, the posterior boundary of this domain is irregular and the domain is somewhat narrower indicating posterior reduction of the *Tc-lim* marked tissues (Figure 4H). In wild-type elongating germbands, additional *Tc-lim1* segmental expression arises that laterally overlaps the segmental *Tc-wg* domains in gnathal and thoracic segments (Figure 4K). At these stages, the ocular-antennal domain of *Tc-lim1* expression splits into a posterior domain that overlaps the antennal *Tc-wg* stripe, and an anterior domain positioned between the ocular and the antennal *Tc-wg* stripes (Figure 4K; white arrowheads). Expression in the ocular-antennal region remains also in *Tc-kni*^RNAi^ germbands, but the domain is not split as in wildtype (upper red arrowhead in Figure 4L) and appears to be fused with the mandibular domain of lateral *Tc-lim1* expression (lower red arrowhead in Figure 4L). In extreme cases this domain is also fused to the maxillary *Tc-lim1* expression domain (Figure 4L and discussed below). This confirms that anterior antennal tissue is properly specified while the parasegmental boundaries of the antennal and mandibular segments are not formed correctly.

**Figure 5:**
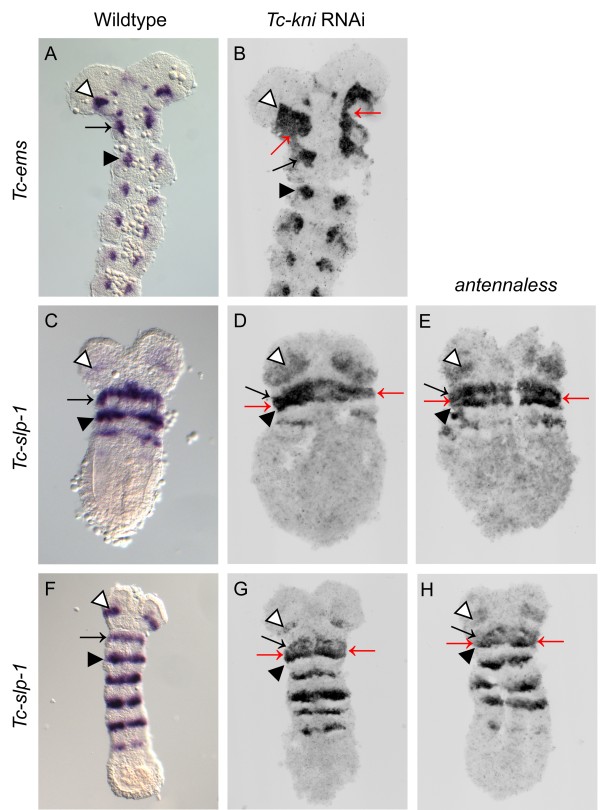
**Expression of *Tc-ems *and *Tc-slp-1 *in wildtype, *Tc-kni *RNAi and *antennaless *embryos.** Panels **B**, **D, E, G and H** represent documentation of fluorescent FastRed stainings of the respective gene and are therefore shown in grayscale. *Tc-ems* expression in the antennal (white arrowhead, panels **A, B**) and mandibular (black arrow, panels **A, B**) segments is abnormally fused in *Tc-kni* RNAi embryos (red arrows in panel **B**; compare to panel **A**). A black arrowhead marks the maxillary domain of *Tc-ems* expression in panels **A, B**. The antennal *Tc-slp-1* expression domain (white arrowhead, panels **C-H**) is often expanded, while the mandibular (black arrow, panels **C-H**) and maxillary (black arrowhead, panels **C-H**) *Tc-slp-1* expression domains are fused (red arrows in panels **D-E, G-H**) in *Tc-kni* RNAi and *antennaless* embryos. Ventral views, and anterior to the top, in all panels.

*Tc-empty-spiracles* (*Tc-ems*) is expressed in a segmentally reiterated pattern during *Tribolium* development [36] in mediolaterally-restricted domains that lie anterior to the *Tc-wg* stripe in each segment [36]. *Tc-ems* expression therefore predominantly marks the anterior portion of each segment. In contrast to wild-type expression, the antennal and mandibular *Tc-ems* segmental domains are fused in *Tc-kni*^RNAi^ embryos (red arrows in Figure 5B), as seen for *Tc-lim1*. This is consistent with defects being restricted to the posterior compartment of the antennal segment leading to the fusion of the antennal and mandibular *Tc-ems* expression domains.

*Tc-sloppy-paired-1* (*Tc-slp-1*) is also expressed in a segmentally reiterated pattern in the developing *Tribolium* head (Figure 5C, F and [25,32]). *Tc-slp-1* expression domains overlap the *Tc-wg* stripe in each head segment, but also extend further into the anterior compartment, as well as a little across the parasegmental boundary into the posterior compartment [25,32]. *Tc-slp-1* expression therefore predominantly marks a posterior portion of the anterior segment compartment. *Tc-slp-1* is expressed in a stripe in the antennal and each gnathal head segment in both *Tc-kni*^RNAi^ and *Tc-kni*^atl^ embryos (Figure 5D, E and G, H), as in wild-type embryos (Figure 5C, F). However, the antennal stripe often appears to broaden (white arrowhead in Figure 5D, E, H), and the distance between the mandibular and the maxillary *Tc-slp-1* stripes is often decreased, completely fusing in lateral regions (red arrows in Figure 5D, E and G, H). These data indicate that overall the *Tc-slp-1* stripes are not dramatically affected by *Tc-kni* RNAi. However, the decreased distance between the mandibular and maxillary stripes could indicate incorrectly specified tissue in the intervening posterior compartment of the mandibular segment.

**Figure 6:**
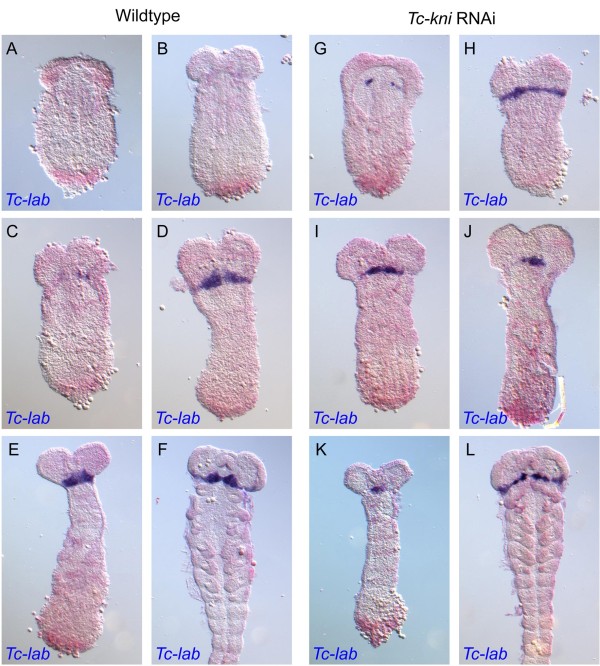
**Expression of *Tc-lab *in wildtype and *Tc-kni *RNAi embryos.***Tc-lab* expression appears prematurely in *Tc-kni* RNAi embryos (panels **G, H, I**; compare to equivalent stage wildtype embryos in panels **A, B, C** respectively). In *Tc-kni* RNAi embryos *Tc-lab* expression is always present but somewhat disturbed, including lack of median enlargement (**H, I, L,** compared to wildtype embryos in panels **D, F**) or lateral reduction (**I, J, K,** compare to wildtype embryos in panels **D, E**). See text for further details. The red background staining in each panel is weak FastRed signal for *Tc-wg*.

A normal complement of bristles and setae in lateral regions of *Tc-kni*^atl^ larval heads implies the retention of intercalary segment derived cuticle in *Tc-kni* knockdown embryos (Figure 1 and [32]). Since *Tc-labial* (*Tc-lab*) is expressed during embryogenesis throughout the presumptive intercalary segment [58], and is required for its formation [32], we used it as a molecular marker for the presence/absence of the intercalary segment. In *Tc-kni* RNAi embryos (Figure 6G-L), *Tc-lab* expression appears prematurely but with similar dynamics as in wildtype (Figure 6A-F). The premature *Tc-lab* expression domain first appears medially as a pair of dots on either side of the median mesoderm (Figure 6G; compare to wild-type in Figure 6A). *Tc-lab* expression then extends into more lateral and medial regions. Expression is not entirely wildtype, as it may form an unusual regular stripe without the typical median expansion (compare Figure 6H, L with D, F), or it may be laterally reduced (Figure 6I, J, K). However, in all instances, the stripe was present indicating that the intercalary segment is present albeit its morphology appears not to be entirely unaffected by adjacent defects.

**Figure 7:**
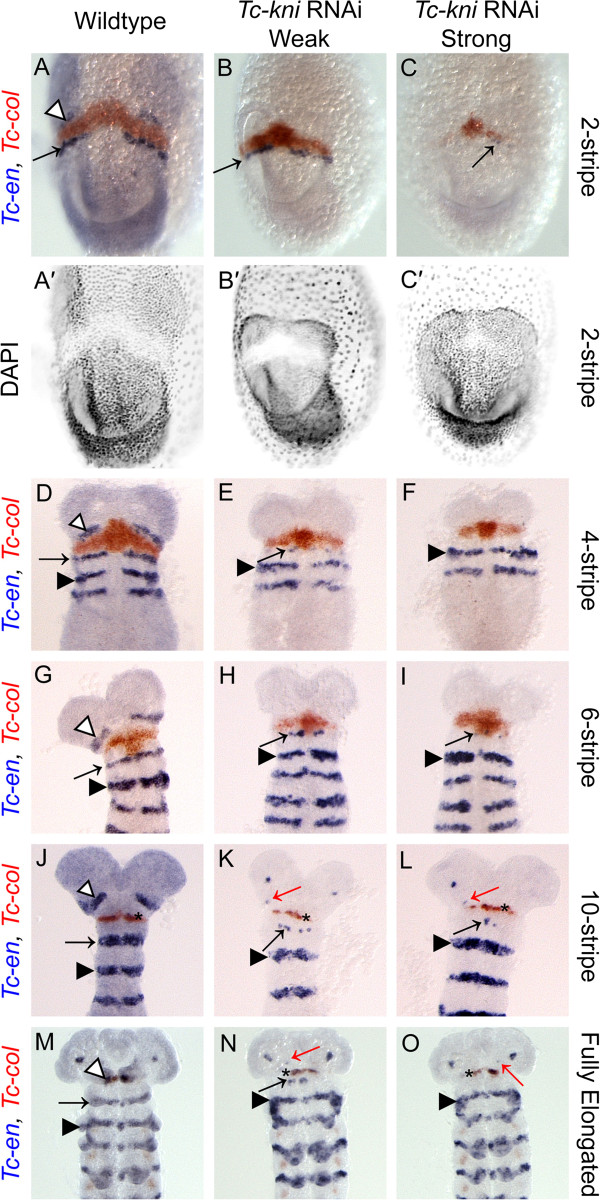
**Expression of *Tc-col *and *Tc-en *in wildtype and *Tc-kni *RNAi embryos.** Abnormalities in *Tc-en* expression in *Tc-kni* RNAi embryos are similar to those seen in Figure 2 and described in the text. The appearance of the *Tc-col* domain in early germband embryos may be slightly delayed following *Tc-kni* RNAi (compare panels **B, C** to panel **A**). Apart from minor irregularities, the *Tc-col* expression domain is not strongly altered in *Tc-kni* RNAi embryos (compare wildtype to *Tc-kni* RNAi embryos in panels **D**-**O**). The co-expression of *Tc-col* and *Tc-en* in a medial region of the head in mid-to-late germband embryos (black asterisk in panels **J-L, N-O**, obscured by antennae in **M**) confirms the identity and presence of intercalary *Tc-en* expression in a *Tc-kni* RNAi background. White arrowheads: *Tc-en* antennal stripe. Black arrows: *Tc-en* mandibular stripe. Black arrowheads: *Tc-en* maxillary stripe. Red arrows: Residual *Tc-en* expression associated with structures at the base of the antennae. Ventral views, and anterior towards the top, in all panels.

We also examined the expression of another marker for the intercalary segment in *Tc-kni*^RNAi^ embryos, the second order regulator *Tc-collier* (*Tc-col*), also called *Tc-knot*. *Tc-col* acts downstream of *Tc-lab* and is required for wildtype expression of *Tc-en* in the intercalary segment [31]. Aside from a slight delay in the initial appearance of the *Tc-col* expression domain in early embryos (Figure 7 compare B, C with A) we found no evidence of strong disruption of *Tc-col* expression in a *Tc-kni*^RNAi^ background (Figure 7). Besides, this staining confirmed the identity, and wildtype appearance, of the intercalary spots of *Tc-en* in late elongating and early fully elongated embryos (black asterisk in panels K, L, N and O in Figure 7).

**Figure 8:**
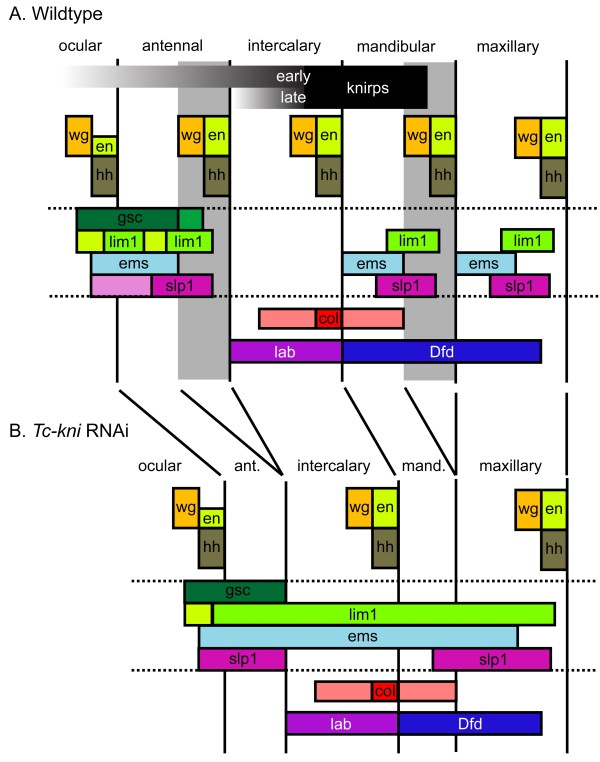
**Schematic diagrams summarizing the expression of *Tribolium *head genes in wildtype and *Tc-kni***^**RNAi **^**embryos.** Changes in marker gene expression following *Tc-kni* RNAi are consistent with a failure to specify and form posterior antennal and mandibular segmental regions (marked in grey in **A**, and deleted in **B**). Deletion of these regions explains the absence of segment polarity gene expression in antennal and mandibular segments, the deletion of posterior regions of the ocular/antennal domains of *Tc-gsc* and *Tc-lim1* at early stages, the reduced width of the *Tc-Dfd* domain, and the reduction in distance between antennal, mandibular and/or maxillary domains of *Tc-lim1*, *Tc-ems* and/or *Tc-slp-1*. Abnormalities in the expression patterns of the genes bounded by the horizontal doted lines are most apparent in lateral regions of the germband. Additional defects affecting the maxillary segment have to be assumed to explain the fusion of respective *Tc-ems* and *Tc-slp* stripes. These could be due to aberrant morphogen signaling of the mandibular parasegment boundary. In *Tc-kni* RNAi, the antennal parasegment boundary is never established while our model assumes that the posterior of the mandibular segment is lost later due to disturbance of the *wg*/*hh*/*en* regulatory loop. Lightly shaded regions represent early aspects of expression that do not persist to later stages. See Figure 10 and text for details.

The *Drosophila* head gap genes lead to the loss of entire adjacent segments but their *Tribolium* orthologues do not fit this definition [36]. Similarly, taken together, our data (summarized in Figure 8) show that *Tc-kni* cannot be considered a canonical head gap gene, because *Tc-kni* function is only required for the posterior parts of the antennal and mandibular segments (the regions shaded grey in Figure 8A). Moreover, *Tc-kni* is not required for the formation of the intercalary segment, which lies between the affected antennal and mandibular segments. Intercalary segment polarity gene expression is instead likely dependent on a conserved pathway involving *Tc-lab* and *Tc-col*[31,32]. Overall, our data confirm that the regulatory networks underlying the establishment of the anterior head segments have diverged significantly between *Tribolium* and *Drosophila*, involving changing roles for *knirps*, *buttonhead* and *empty spiracles* homologues [36].

### The *Tc-kni* phenotype is due to a failure to correctly specify cell fates

We decided to check whether missing head regions in *Tc-kni* knockdown and mutant embryos were lost due to a failure to maintain already specified tissue - which might be indicated by high levels of cell death - or through the failure to specify cells to their correct developmental fate. Using TUNEL staining, we did not detect any apoptosis in either the blastoderm (see Additional file 3) or early germband stages (see Additional file 4) in *Tc-kni*^RNAi^ embryos. In later germband stages there were a few apoptotic cells detectable in the head (as well as the posterior growth zone), but no more than in wild-type embryos, and there was no specific pattern of apoptosis that would indicate the loss of the tissues in question, namely the posterior portions of the antennal and mandibular segments (see Additional files 3 and 4). Thus, the lack of antennae and mandibles in *Tc-kni*^RNAi^ and *Tc-kni*^atl^ larvae is not due to tissue degeneration, but rather the failure to specify the respective segmental regions.

### The posterior border of the anterior *Tc-kni* expression domain is regulated by *Tc-even-skipped*

**Figure 9:**
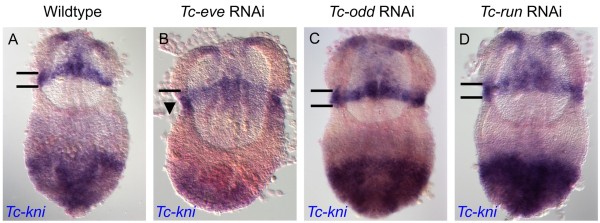
***Tc-kni *is regulated by *Tc-eve*.** The *Tc-kni* head expression domain, which covers the anterior compartment of the mandibular segment (bounded by black horizontal lines in panels **A, C, D**) extends posteriorly (arrowhead in panel **B**), in a *Tc-eve* RNAi background, but not in *Tc-odd* RNAi (panel **C**) or *Tc-run* RNAi (panel **C**) embryos. This is consistent with *Tc-eve* expression in the posterior compartment of the mandibular segment setting the posterior boundary of the *Tc-kni* head expression domain.

In the *Tribolium* blastoderm, expression of *Tc-kni* and the pair rule genes occurs at the same time, opening the possibility *Tc-kni* could be regulated by pair rule genes. Indeed, in *Tc-eve*^RNAi^ blastoderm embryos, we found that the anterior domain of *Tc-kni* expression expanded into more posterior regions (Figure 9: compare panel B to panel A). In contrast, expression defects in this *Tc-kni* mandibular domain were not seen in *Tc-odd*^RNAi^ or *Tc-run*^RNAi^ germbands (Figure 9: compare panels C and D with A). This is consistent with the first pair-rule and/or segmental stripe of *Tc-eve* expression being required to set the posterior boundary of mandibular *Tc-kni* expression.

### Head and trunk segmentation gene networks cooperate to establish the mandibular segment in a different way in *Tribolium* and *Drosophila*

**Figure 10:**
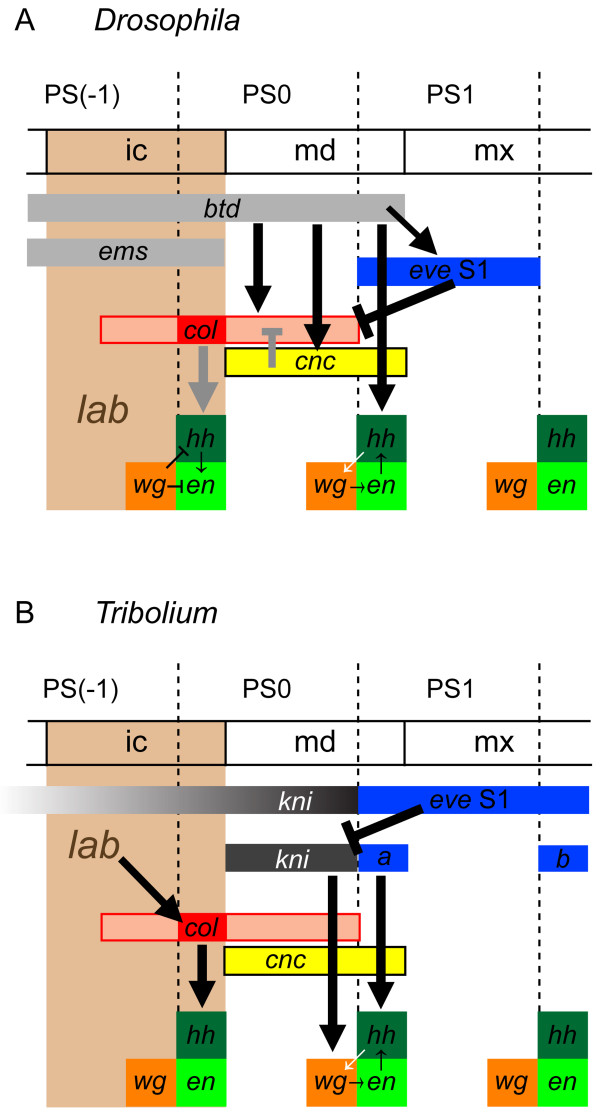
**The genetic interactions that help establish the PS0/PS1 parasegmental boundary in *Drosophila *and *Tribolium*.** In *Drosophila* (**A**), expression of the mandibular *engrailed* (*en*) stripe is dependent on the activity of *buttonhead* (*btd*) [9]. In contrast, in *Tribolium* (**B**), expression of the mandibular *Tc-en* stripe is dependent on the combined activity of *Tc-kni* in the anterior compartment (PS0) (this study) and *Tc-eve* in the posterior compartment (PS1) [24]. Cap‘n’collar (Cnc) may limit positive regulation of *hh* by *col* to the intercalary segment by physically interacting with Col in the mandibular segment [22]. The interactions between the segment polarity genes in the mandibular segment in *Tribolium* are assumed from what is known in *Drosophila*[13]*.* Black symbols: Possible direct genetic interactions. Gray symbols: Direct interactions shown in [22].

In *Drosophila*, the anterior head gene regulatory network (through *btd*, *col* and *cnc*) and the gnathal/trunk regulatory gene network (through *eve*) cooperate to pattern aspects of the mandibular segment (Figure 10A and [11,12]). In this study we provide evidence that this is also true in *Tribolium*. Both *Tc-eve* and *Tc-kni* RNAi lead to the failure to properly maintain mandibular *Tc-en* stripe expression and as a result to loss of the mandible itself (this study and [24,43]). In our model (Figure 10B) *Tc-kni* contributes to the activation of mandibular *Tc-wg* expression. This interaction could be direct because the respective expression patterns overlap. *Tc-eve* in turn could be directly involved in activating *Tc-hh* and *Tc-en* within its anteriormost expression domain. Subsequently, the interactions of the Wnt and hh pathways ensure maintenance of the parasegement boundary. In the absence of *Tc-kni* expression, *Tc-eve* still partially initiates *Tc-en/Tc-hh* expression in the posterior compartment, which leads to partial initiation of *Tc-wg* in the anterior adjacent cells (through Hedgehog signaling). However, absence of *Tc-kni* leads to failure to maintain *Tc-wg* expression, and as a result failure to maintain *Tc-en* expression in posterior adjacent cells (through Wingless signaling). Interestingly, in *Drosophila*, the maintenance of *en* and *wg* expression in the mandibular segment appears more interdependent in dorsal regions [13]. The same might also be true in *Tribolium*, since both *Tc-en* and *Tc-wg* expression is maintained more often in medial (i.e. ventral) regions in *Tc-kni* mutant and knockdown embryos. Taken together, it appears to be a conserved feature of insects that two systems cooperate in patterning the mandible, but the molecular details of this cooperation have diverged significantly between *Drosophila* and *Tribolium* (Figure 10).

## Conclusions

In this study we show that anterior regions of the antennal and mandibular segment, as well as the intervening intercalary segment, are correctly specified in the absence of *Tc-kni* function. Moreover, we identify key differences in the role of *Tc-kni* in setting up the non-adjacent antennal and mandibular parasegment boundaries. We identify the pair-rule gene *Tc-even-skipped* as a potential positive regulatory input that enables the initial specification (but not maintenance) of the mandibular parasegmental boundary in the absence of *Tc-kni* function. We also present the first evidence that, as in *Drosophila*, head and trunk gene regulatory networks cooperate to specify the mandibular segment. However, our data, and those of others, point to significant divergence in the molecular interactions involved in mandibular patterning between *Drosophila* and *Tribolium*.

A recent study of the expression and function of head patterning genes in the hemimetabolous insect *Oncopeltus fasciatus* suggests that the developmental gene networks operating in *Tribolium castaneum* more closely resemble the ancestral insect condition, perhaps not surprisingly given the highly derived *Drosophila* larval head [59]. Indeed, recent data from a myriapod suggest that the developmental genetic basis of *Tribolium* larval head development might even closely resemble the ancestral arthropod condition [60,61]. It will be interesting to see whether studies on other arthropods reveal an ancestral role for *knirps*-family homologues in head segmentation, which would imply *ems* and *btd* have usurped the role of *knirps*, and potentially other genes, in the lineage leading to *Drosophila*.

## Methods

### *Antennaless* mutant analysis

The *antennaless* mutant line was maintained as described by Berghammer *et al.*[62] and Maderspacher *et al.*[54]. The separation of offspring from single pair crosses was performed after three and six days of egg laying at 32°C and 25°C respectively. Cuticle preparations of mutant larvae were prepared as previously described [54]. Genomic DNA was extracted from individual L1 *antennaless* mutant and wildtype larvae by first removing flour and chorion by bleaching egg collections two times for four minutes in 100% bleach. Embryos were washed three times with extraction buffer (without proteinase K - see below), put individually into 0.5 ml cups and frozen at −20°C. 10 μl of extraction buffer was added (25 mM NaCl, 10 mM Tris pH8.0, 1 mM EDTA, 200 μg/ml proteinase K) and the embryos macerated with a pipette tip. After 90mins at 42°C, the preparations were shortly spun down and proteinase K was inactivated by three minutes at 95°C. Debris was spun down for two minutes at 14000 rpm and 8 μl of supernatant was transferred to new tubes. 4 μl were used for a 20 μl PCR reaction. The three *Tc-kni* exons were amplified by PCR using the following four primer combinations: ex1 fw 5′-ACATTCCCCACCATTGAAATCACA-3′; ex1 rev 5′-GGGTTAAGTTTCTCGGTATTGGGACTA-3′; ex2 fw 5′-CCTGTAATGTGTACAGTCACGAGCAG-3′; ex2 rev 5′-ATTCTTGCATCGGCCGAAGTTTACGT-3′; ex3A fw 5′-CGGAAGCTCTGTCAAACAATAATCTCA-3′; ex3A rev 5′-TCCAGGAACACCCGCTTGTTGA-3′; ex3B fw 5′-CGCCGACGTTTCTACCTCCTCA-3′; ex3B rev 5′-TCGACGCTAATAGCTGCCATCATC-3′. Sequencing of the PCR products was performed by Macrogen (Korea).

### Expression analysis

Fixation of embryos and enzymatic single and double *in situ* hybridizations were carried out according to established protocols [63]. For double *in situ* hybridizations Fastred ® (Sigma) was sometimes used (e.g. for *Tc-wg*) in place of INT/BCIP (Roche 11-681-460-001) [63]. Probes for *in situ* hybridization were prepared using either the Digoxigenin RNA Labeling Kit or Fluorescein RNA Labeling Kit (Roche Applied Science, Mannheim) following established protocols and the manufacturers instructions [63]. The DNA templates used for RNA probe production were in some cases (i.e. for probes detecting *Tc-ems*, *Tc-slp-1*, *Tc-wg*, *Tc-eve*, *Tc-gsc*, *Tc-hh*, *Tc-lab*, *Tc-lim1*, *Tc-odd*, *Tc-run*) produced by PCR-amplifying DNA fragments of the gene of interest from clones using appropriate vector primers (T3, T7, SP6). In other cases (i.e. for probes detecting *Tc-en*, *Tc-col*, *Tc-Dfd*) clones containing a fragment of the gene of interest were used directly as templates following 5′ linearization using an appropriate restriction enzyme. Clones and information are available on request. Following *in situ* hybridizations, nuclei in blastoderm and early germband embryos were sometimes counterstained using Hoechst 33258 (Additional file 2), Hoechst 33342 (Additional file 3) or DAPI (Figures 1, 2 and 7).

### Parental RNAi

Adult or pupal parental RNAi was carried out using established protocols [64]. dsRNA was produced using the T7 and SP6 MEGAscript High Yield Transcription Kits (Ambion). Template DNA was either PCR-amplified using the vector insert flanking primers T7: 5′-TAATACGACTCACTATAGG-3′ and T7-Sp6: 5′-TAATACGACTCACTATAGGATTTAGGTGAACACTATAGA-3′) or by using a stock of the linearized plasmid. In this case the antisense and sense strands were amplified separately, and later the ssRNA combined in equimolar amounts. A concentration of between 2 and 4.3 μg/μl of *Tc-kni* dsRNA was injected in each experiment, since this concentration range has been previously shown to consistently produce fully penetrant *Tc-kni* RNAi phenotypes [43]. To knockdown *Tc-eve*, *Tc-odd* and *Tc-runt* concentrations of between 2 and 3.5μg/μl of dsRNA were used.

### TUNEL staining

Fixed embryos stored in methanol at −20°C were gradually rehydrated by washing in 70% methanol/PBT, then 50% methanol/PBT, then 30% methanol/PBT and finally 100% PBT (PBS + 0.1% (v/v) Tween-20). Embryos were then incubated for six minutes in 1 ml of PBT + 0.5 μl proteinase K (15 mg/ml) and subsequently washed several times in PBT. Embryos were then post-fixed in 1 ml of PBT + 125 μl of formaldehyde (37%) for 20 minutes, before being washed three times in PBT. At this point, positive control embryos were washed three time in DNaseI buffer (40 mM Tris–HCl, pH7.5, 0.1 mM dithiothreitol, 6 mM MgCl_2_), incubated for 30 minutes at 37°C in DNaseI buffer with 0.06 U DNaseI per microlitre of buffer, before being washed several times in PBT. All embryos were then incubated for 20 minutes in 0.1% sodium borohydride. During incubation, the embryos were gently shaken several times. The sodium borohydride was then removed by repeated washing with TdT buffer (140 mM cacodylic acid, 1 mM cobalt chloride, 30 mM Tris–HCl, pH7.2). Embryos were then incubated at 37°C in TdT buffer containing 20 μM DIG-UTP and 0.3 U/μl terminal deoxynucleotidyl transferase (TdT) (Sigma). In the case of negative control embryos, the TdT was omitted. All embryos were then washed three times for five minutes in TBST (140 mM NaCl, 2.7 mM KCl, 25 mM Tris–HCl pH7.4, 0.1% Tween-20) at room temperature, before being incubated at 70°C for 20 minutes in TBST. Embryos were then washed three times for five minutes in PBT, before being incubated in PAS (PBT with 10 mg/ml bovine albumine (BSA) and 2% sheep serum) for one hour. Embryos were then incubated in PAS with anti-Dig antibody (1:2000) for one hour. This was followed by several washes in PBT for a total of two hours. Embryos were stored overnight at 4°C in PBT before being washed for 30 minutes at room temperature in PBT prior to NBT/BCIP staining. The staining was stopped by repeated washing in PBT and the embryos stored at 4°C in 1 ml of PBT + 125 μl of formaldehyde (37%).

### Microscopy

Most *in situ* hybridization stained preparations were imaged on an Axioplan 2 photomicroscope (Carl Zeiss Vision GmbH, Jena) using a polarized light (DIC) filter with low Normaski contrast (ImageProPlus, Version 5.0 .2.9, MediaCybernetics). For clear detection of the fluorescence signal of the Fastred® color reaction the filter set No. 43 (Cy3) from Zeiss, and a mercury vapor lamp HBO100 as a light source, was used. For the detection of Hoechst 33258 and Hoechst 33342 signal the filter set No. 49 from Zeiss (DAPI filter), and a mercury vapor lamp as the light HBO100 source, was used. A few *in situ* hybridization stained preparations were imaged on a Leica MZ16F epifluorescence stereoscope with a DFC300FX digital camera (images in Figures 2 and 7). Larval cuticles were imaged using a confocal laser-scanning microscope (LSM 510, Zeiss) and processed as described [65].

### Availability of supporting data

The data set supporting the results of this article is included within the article (and its additional files).

## Competing interests

The authors declare that they have no competing interests.

## Authors’ contributions

AP performed experiments, analyzed the data and wrote the paper, DG, JS, FR and GO performed experiments and analyzed the data, JS supervised experiments and analyzed the data, AG drafted the paper, MK identified the *atl* mutant, and GB designed the study and wrote the paper. All authors read and approved the final manuscript.

## Supplementary Material

Additional file 1 **The sensitivity of *Tc-kni *RNAi head and abdominal larval phenotypes to temperature.** (**A, B**) Head phenotypes are more severe at higher temperatures. Note the higher frequency of strong head phenotypes (up to ~90% vs. circa 70%) and lower frequency of weak head phenotypes (0% at some time points at 32°C) following *Tc-kni* RNAi carried out at 32°C compared to 25°C. Larvae exhibiting strong head phenotypes lacked both antennae and mandibles, larvae exhibiting medium phenotypes possessed at least one antenna and larvae exhibiting weak phenotypes exhibited at least one antenna and mandibles. (**C**) The frequency of larval head phenotypes decreases with time post *Tc-kni* dsRNA injection. (**D, E**) In contrast to head phenotypes, abdominal phenotypes showed an unusual reverse sensitivity with respect to temperature and time following *Tc-kni* RNAi injection. Abdominal phenotypes were more common following *Tc-kni* parental RNAi carried out at 25°C compared to 32°C (compare height of blue bars in panel **D** vs. panel **E**). Abdominal phenotypes showed the unusual characteristic of increasing in frequency with time post *Tc-kni* dsRNA injection. Click here for file

Additional file 2 ***Tc-kni* is not expressed in *antennaless* embryos.** Wildtype and *antennaless* blastoderm (panels **A-D**) and early germband (panels **E-H**) embryos co-stained with a mix of *Tc-kni* and *Tc-cad* probes detected with the same colour reaction. A probe against *Tc-cad* was used to control against the possibility that the absence of *Tc-kni* signal in *antennaless* embryos was due to technical problems. Blastoderm embryos were stained with Hoechst 33258 (**A’-D’**) in order to identify similar stage embryos. In *antennaless* blastoderm embryos, a block of signal (bounded by white lines in panels **A, C**) corresponding to the anterior head *Tc-kni* expression domain is missing, whereas the posterior domain of *Tc-cad* expression is detected. In *antennaless* early germband embryos the anterior mandibular stripe of *Tc-kni* expression (black arrowhead in panels **E, G**) is missing, whereas the posterior growth zone domain of *Tc-cad* expression is detected. Similar experiments using a *Tc-otd* probe as control proved that the posterior *Tc-kni* expression domain is also missing in *antennaless* blastoderm and germband embryos (data not shown). Panels **A-D’:** Lateral views, anterior to the left. Panels **E-H:** Ventral views, anterior to the top. Click here for file

Additional file 3 **TUNEL stained wildtype and *Tc-kni* RNAi blastoderm stage embryos.** DAPI staining (**A’-F**’) was used to identify wildtype and *Tc-kni* RNAi blastoderm embryos of similar stages. No apoptotic nuclei were observed in wildtype or *Tc-kni* RNAi blastoderm embryos. Click here for file

Additional file 4 **TUNEL stained wildtype and *Tc-kni *RNAi germband stage embryos.** Apoptotic nuclei were not observed in wildtype or *Tc-kni* RNAi early germband stage embryos (**A-F**). A few apoptotic nuclei (arrowheads in panels **G-J**) were observed in mid-elongation (**G-H**), late-elongation (**I-J**) and fully elongated (**K-L**) wildtype and *Tc-kni* RNAi germband embryos. However, levels of apoptotic nuclei were no higher in *Tc-kni* RNAi germband embryos when compared to controls, and apoptotic nuclei were not concentrated in regions within which the antennal and mandibular segments should or would develop. Note that in some cases (panels **C, E, G**, **I, N**) TUNEL reactions were developed for much longer than needed to detect apoptotic nuclei, leading to background staining. Note that apoptotic nuclei can nevertheless be distinguished from background (e.g. panels **G, I**). Click here for file
