# Supplementary figures and images for: Tc-knirps plays different roles in the specification of antennal and mandibular parasegment boundaries and is regulated by a pair-rule gene in the beetle Tribolium castaneum
Source: BMC Dev Biol. 2013 Jun 18;13:25. doi: 10.1186/1471-213X-13-25 (PMC3698154; doi:10.1186/1471-213X-13-25)

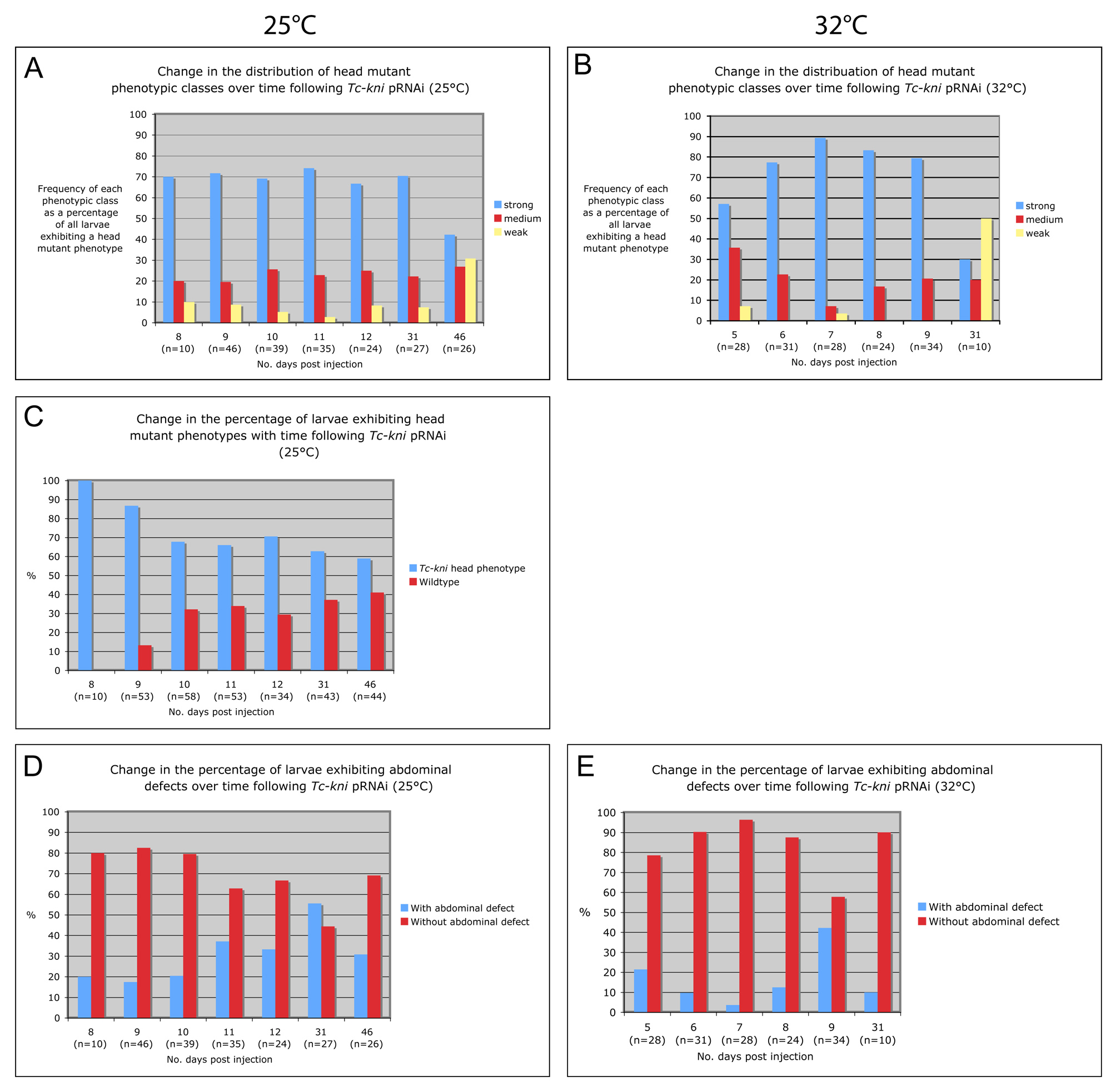

Supplement: Additional file 1 — The sensitivity of Tc-kni RNAi head and abdominal larval phenotypes to temperature. (A, B) Head phenotypes are more severe at higher temperatures. Note the higher frequency of strong head phenotypes (up to ~90% vs. circa 70%) and lower frequency of weak head phenotypes (0% at some time points at 32°C) following Tc-kni RNAi carried out at 32°C compared to 25°C. Larvae exhibiting strong head phenotypes lacked both antennae and mandibles, larvae exhibiting medium phenotypes possessed at least one antenna and larvae exhibiting weak phenotypes exhibited at least one antenna and mandibles. (C) The frequency of larval head phenotypes decreases with time post Tc-kni dsRNA injection. (D, E) In contrast to head phenotypes, abdominal phenotypes showed an unusual reverse sensitivity with respect to temperature and time following Tc-kni RNAi injection. Abdominal phenotypes were more common following Tc-kni parental RNAi carried out at 25°C compared to 32°C (compare height of blue bars in panel D vs. panel E). Abdominal phenotypes showed the unusual characteristic of increasing in frequency with time post Tc-kni dsRNA injection. [file 1471-213X-13-25-S1.jpeg]

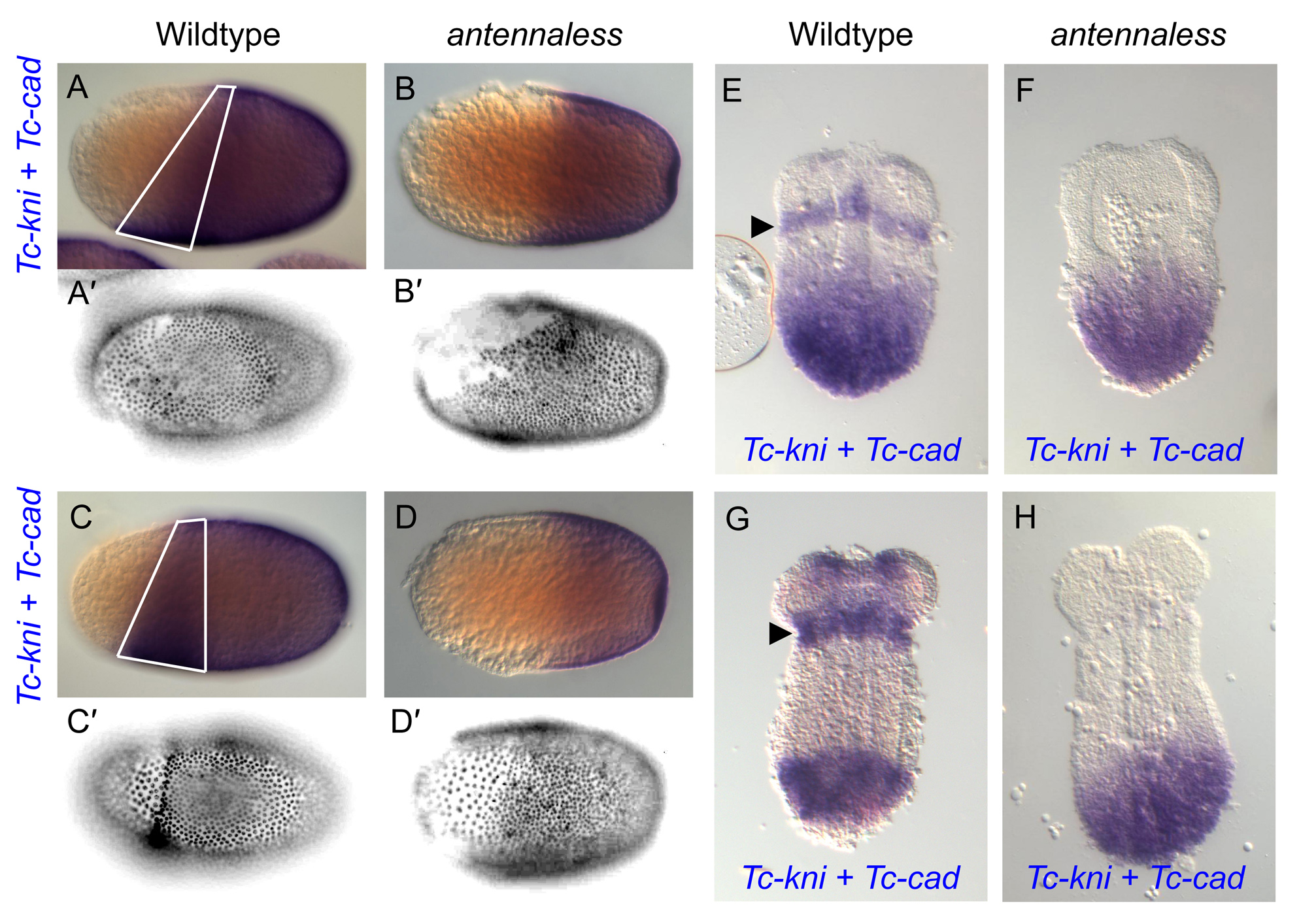

Supplement: Additional file 2 — Tc-kni is not expressed in antennaless embryos. Wildtype and antennaless blastoderm (panels A-D) and early germband (panels E-H) embryos co-stained with a mix of Tc-kni and Tc-cad probes detected with the same colour reaction. A probe against Tc-cad was used to control against the possibility that the absence of Tc-kni signal in antennaless embryos was due to technical problems. Blastoderm embryos were stained with Hoechst 33258 (A’-D’) in order to identify similar stage embryos. In antennaless blastoderm embryos, a block of signal (bounded by white lines in panels A, C) corresponding to the anterior head Tc-kni expression domain is missing, whereas the posterior domain of Tc-cad expression is detected. In antennaless early germband embryos the anterior mandibular stripe of Tc-kni expression (black arrowhead in panels E, G) is missing, whereas the posterior growth zone domain of Tc-cad expression is detected. Similar experiments using a Tc-otd probe as control proved that the posterior Tc-kni expression domain is also missing in antennaless blastoderm and germband embryos (data not shown). Panels A-D’: Lateral views, anterior to the left. Panels E-H: Ventral views, anterior to the top. [file 1471-213X-13-25-S2.jpeg]

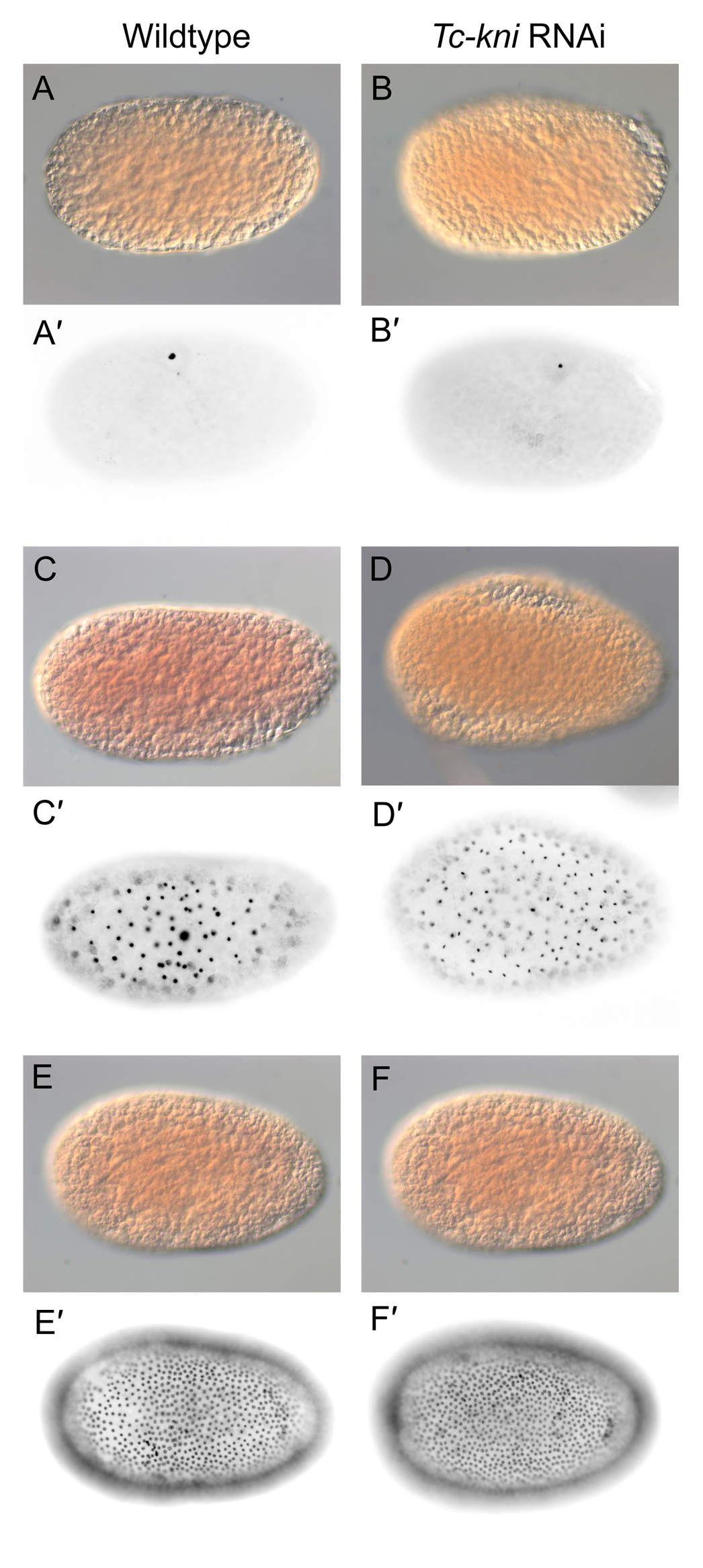

Supplement: Additional file 3 — TUNEL stained wildtype and Tc-kni RNAi blastoderm stage embryos. DAPI staining (A’-F’) was used to identify wildtype and Tc-kni RNAi blastoderm embryos of similar stages. No apoptotic nuclei were observed in wildtype or Tc-kni RNAi blastoderm embryos. [file 1471-213X-13-25-S3.jpeg]

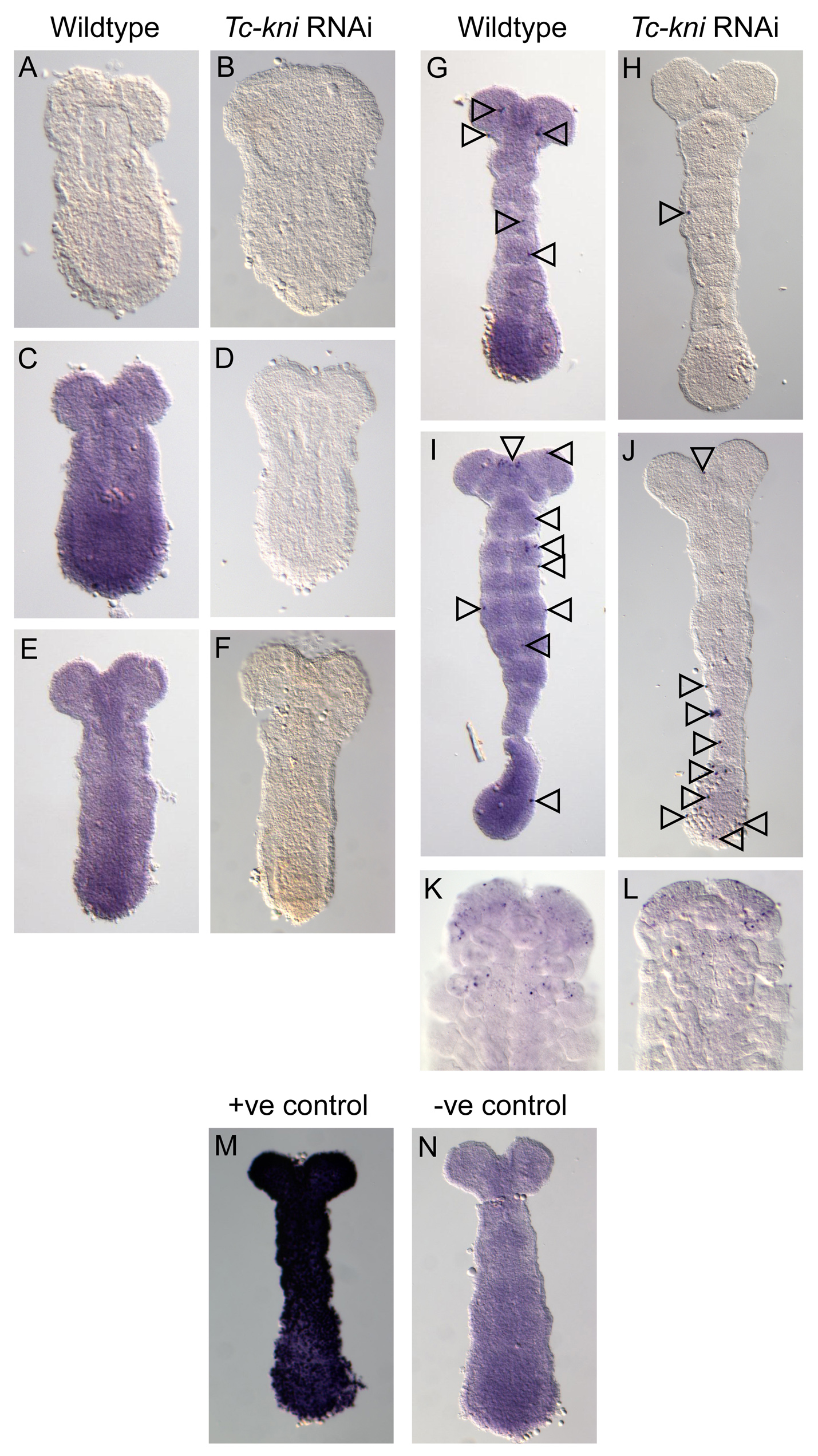

Supplement: Additional file 4 — TUNEL stained wildtype and Tc-kni RNAi germband stage embryos. Apoptotic nuclei were not observed in wildtype or Tc-kni RNAi early germband stage embryos (A-F). A few apoptotic nuclei (arrowheads in panels G-J) were observed in mid-elongation (G-H), late-elongation (I-J) and fully elongated (K-L) wildtype and Tc-kni RNAi germband embryos. However, levels of apoptotic nuclei were no higher in Tc-kni RNAi germband embryos when compared to controls, and apoptotic nuclei were not concentrated in regions within which the antennal and mandibular segments should or would develop. Note that in some cases (panels C, E, G, I, N) TUNEL reactions were developed for much longer than needed to detect apoptotic nuclei, leading to background staining. Note that apoptotic nuclei can nevertheless be distinguished from background (e.g. panels G, I). [file 1471-213X-13-25-S4.jpeg]
